# Identification and Nutritional, Bioactive, and Antioxidant Evaluation of a Novel Mushroom Species, *Inonotus chrysosporus*

**DOI:** 10.3390/jof12070517

**Published:** 2026-07-15

**Authors:** Feifei Song, Zhiping Li, Nemat O. Keyhani, Yusheng Zhang, Mengjia Zhu, Lin Zhao, Ligong Shen, Dewei Su, Junzhi Qiu

**Affiliations:** 1 State Key Laboratory of Agricultural and Forestry Biosecurity, College of Life Sciences, Fujian Agriculture and Forestry University, Fuzhou 350002, China; flyinthesky-feifei@163.com (F.S.); yushengzhang2001@163.com (Y.Z.); zhumengjia_0529@163.com (M.Z.); linzh1995@126.com (L.Z.); 2 College of Horticulture, Fujian Agriculture and Forestry University, Fuzhou 350002, China; lizhiping20122022@163.com; 3 Department of Biological Sciences, University of Illinois, Chicago, IL 60607, USA; keyhani@uic.edu; 4 Analysis and Testing Center, Fujian Agriculture and Forestry University, Fuzhou 350002, China; slg@fafu.edu.cn; 5 National Engineering Research Center of Juncao Technology, Fujian Agriculture and Forestry University, Fuzhou 350002, China

**Keywords:** *Inonotus chrysosporus*, nutritional components, bioactive constituents, antioxidant activity

## Abstract

*Inonotus* is a globally distributed genus within the Hymenochaetales, with certain species widely used in traditional herbal medicine. Here, a new species, *I. chrysosporus*, was identified as a sister clade to *I. hispidus* based on morphological, molecular, and vegetative incompatibility characteristics. The growth of *I. chrysosporus* on two substrate formulations—(1) 60% (wt:wt) *Dicranopteris dichotoma*, mixed with 18% corncob, 20% wheat bran, 1% gypsum, and 1% lime (GS), and (2) 78% sawdust wood chips (SS)—was compared in terms of the accumulation of bioactive components and the corresponding biological activities. On both substrates, *I. chrysosporus* exhibited high nutritional value, with high protein and mineral contents, low fat content, and a high proportion of medicinal amino acids. However, significant variations in the profiles of active constituents were observed, with cultivation on the GS formulation promoting the accumulation of bioactive polysaccharides, whereas growth on SS resulted in the accumulation of polyphenols. Aqueous extracts of *I. chrysosporus* mushrooms derived from both growth substrates exhibited potent antioxidant activities. Correlation analyses indicated that polysaccharide content was positively associated with ·OH scavenging activity and that polyphenol content was correlated with strong DPPH radical scavenging activity and high ferric-reducing antioxidant power (FRAP). This study provides a foundation for the comprehensive development and utilization of the newly described *I. chrysosporus* species.

## 1. Introduction

*Inonotus*, one of the largest genera in the family Hymenochaetaceae, typified by *Inonotus hispidus* (Bull.) P. Karst., occurs mainly as a parasite or saprophyte on a wide range of woody substrates [1]. Species of this genus have annual to perennial, resupinate, effused-reflexed, or pileate basidiomata, with yellowish to brown pilei that are hispid, velutinate to rough, or glabrous; a brown pore surface; and a homogeneous, brown, corky context. Certain *Inonotus* species are widely used in traditional medicine to treat a variety of ailments. *Inonotus hispidus*, commonly known as the shaggy bracket mushroom, is widely distributed in North America and Asia and has been used for millennia in traditional Chinese medicine, where it is known as *Sanghuang* [2]. In the Chinese pharmacopeia, the wild fruiting body, considered inedible due to its tough texture and typically found protruding from the bark of living or dying hardwood trees, is traditionally used in teas and extracts to treat a wide variety of diseases, including dyspepsia, gastric ulcers, diabetes, and cancer [3,4]. A broad range of biological activities has been demonstrated for *I. hispidus* extracts, including antioxidant [5], anti-inflammatory [6], antiproliferative (antitumor) [7], hypouricemic [8], hypolipidemic [9], hypoglycemic [10], and immunomodulatory activities [11].

However, wild *Inonotus* resources have experienced dramatic declines owing to excessive harvesting and habitat loss. While artificial cultivation is possible, major constraints include low productivity and a lack of information concerning growth substrates, which can critically affect the quality and yield of fungal biomass, particularly with respect to bioactive compound content. Aside from saprophytic growth, many mushrooms utilize woody substrates containing cellulose and hemicellulose through the production of various extracellular enzymes. Therefore, different growth substrates can significantly influence fruiting body development and the bioactive profiles/biological efficiency of edible fungi [12]. Thus, the isolation of wild strains with potentially more robust genetic backgrounds, together with the selection of suitable cultivation substrates, are critical steps toward facilitating the industrial-scale cultivation of this genus.

Here, both morphological and molecular data were employed to identify an environmental isolate (wild strain) of *Inonotus*, which is designated here as a new species, *I. chrysosporus*. The effects of extracts from *I. chrysosporus* mushrooms grown on two different cultivation substrates were then tested in relation to the accumulation of bioactive components and key antioxidant activities to determine how these cultivation conditions influence the medicinal value of the mushroom and inform strategies for improved artificial cultivation. The nutrient composition and bioactive components of fruiting bodies were analyzed, with the free radical scavenging and antioxidant activities of aqueous extracts of the fungus examined using a panel of assays. The different cultivation conditions resulted in significant differences in the range of bioactive compounds produced by the mushroom, highlighting the importance of these parameters when evaluating the medicinal value of the resulting products. These results provide an empirical basis for the comprehensive development and utilization of the newly isolated *I. chrysosporus* species.

## 2. Materials and Methods

### 2.1. Test Strains, Fungal Isolation, and Culture Studies

The specimen (designated SH211; GenBank accession numbers: PZ413120 for the 28S rRNA gene and PZ413114 for the ITS region) was collected in Fuzhou, Fujian, and deposited in the Fungarium (HMAS) at the Institute of Microbiology, Chinese Academy of Sciences (CAS). Isolates MS1 (*Sanghuangporus sanghuang*) and MS4 (*Sanghuangporus vaninii*) were obtained from the Mycological Research Center of Fujian Agricultural and Forestry University, China [13,14]. Isolate SH623 (*Inonotus hispidus*) was obtained from the National Engineering Research Center of JUNCAO Technology, Fujian Agricultural and Forestry University, China [15]. The taxonomic data for the new taxon have been submitted to MycoBank.

Environmentally harvested tissue blocks containing the internal tissues of the fruiting bodies of the wild strain SH211 were cut and quickly transferred to PDA medium and incubated at 25 °C in the dark. The resulting pure culture was used as the inoculum for the experiments described below. The original wild specimens were not adequately preserved or photographed at the time of collection. For macro- and micromorphological characterization, isolates were cultured on PDA plates and in fruiting bags (the grass substrate formulation is detailed below).

The growth antagonism of the strain was tested against the strains SH623, MS1, and MS4, as described in [16]. For each test plate, strain SH211 was inoculated at one-third of the plate diameter, while an equal amount of the corresponding test strain was inoculated at the opposite position on the Petri dish. Plates were incubated at 28 °C in the dark and monitored for 7 d. Experiments were repeated three times.

### 2.2. Morphological Analyses

All morphological descriptions and the taxonomic identification of the new species were based on cultivated fruiting bodies. Color designations were based on the color charts described in [17]. For micromorphological studies, dried tissue fragments were observed using a microscope (DM2500, Leica, Wetzlar, Germany) after rehydration in 5% KOH. All microscopic measurements were performed using the proprietary software integrated with the microscope. A minimum of 40 basidiospores and 20 basidia were measured. The abbreviations are defined as follows: L = mean basidiospore length; W = mean basidiospore width; Q = range of the L/W ratio observed among the examined specimens; n = total number of basidiospores measured. Colony and mycelial morphologies were recorded after inoculation on PDA plates. During inoculation, coverslips were inserted into the agar at a 45° angle about 1.5 cm from the inoculation site to allow the mycelium to grow onto the glass; the growing mycelial morphology was then monitored using a microscope [18].

### 2.3. Molecular Identification and Phylogenetic Analysis

Genomic DNA was extracted from fresh mycelium of strain SH211 using the Rapid Fungi Genomic DNA Isolation Kit (Sangon Biotech, Shanghai, China) following the manufacturer’s instructions. The nuclear ribosomal ITS region and the nuclear ribosomal nLSU gene were amplified using the primers ITS5/ITS4 [19] and LR0R/LR5 [20], respectively. PCR reactions were conducted as described in [21]. PCR amplicons were separated by 1% agarose gel electrophoresis and then sent to Shanghai Personal Gene Technology Co., Ltd. (Shanghai, China) for sequencing. Sequences were compared to the NCBI Nucleotide Database using BLAST searches. Sequences from *Phellinus populicola* and *Phellinus laevigatus* were selected as the outgroup. ITS and nLSU sequences were aligned and adjusted separately using MEGA 11. Then, PhyloSuite was used to concatenate and construct a phylogenetic tree using Maximum-Likelihood (ML) and Bayesian Inference (BI) analyses. The consensus tree was generated using FigTree (version 1.4.4). Bootstrap support values (≥70% for ML, ≥0.50 for BI) are shown above the corresponding nodes. Detailed specimen information and GenBank accession numbers are listed in Table A1 of the Appendix A.

### 2.4. Cultivation of I. chrysosporus Fruiting Bodies

The culture media used were as follows: (1) grass substrate (GS) formulation: *Dicranopteris dichotoma* 60% (wt:wt), corncob 18%, bran 20%, lime 1%, gypsum 1%, and water content 63%; and (2) sawdust substrate (SS) formulation: wood chips 78%, bran 20%, lime 1%, gypsum 1%, and water content 63%. The materials for cultivation were thoroughly mixed with water, divided into polypropylene bags, sterilized at 121 °C for 3 h, cooled, and then inoculated with *I. chrysosporus* (SH211) in an ultraclean workbench (600 g of medium with 25 g of mycelia). The bags were transferred to a culture room at 25–28 °C and incubated in the dark. The mycelium was monitored for signs of contamination, and after full colonization (>90%), the bags were transferred to a mushroom chamber and maintained under the following environmental conditions: temperature = 25–32 °C; humidity = 80–90%; astigmatic illumination; and good air circulation. After post-ripening (approximately 5–30 d), the bags were sliced to permit fruiting body formation. Fruiting bodies were harvested when growth had stopped and surface spores were observed (approximately 7–20 d). Following harvest, the fruiting bodies were oven-dried at 50 °C for 2 h and then at 60 °C until constant weight was achieved (approximately 24 h); they were then ground into powder and stored in sealed polyethylene bags prior to analysis. The dried fruiting bodies used for morphological observation were designated as the holotype and permanently deposited in HMAS.

### 2.5. Nutrient Analysis of I. chrysosporus Fruiting Bodies

The contents of total protein, fat, crude ash, and amino acids in the fruiting bodies were determined. Protein content was determined using the Kjeldahl method according to GB 5009.5-2016 [22]. Fat content was determined using the Soxhlet extraction method according to GB 5009.6-2016 [23]. Total ash content was determined using the high-temperature burning method, following GB 5009.4-2016 [24]. Amino acid contents were analyzed as described in [25]. Briefly, the ground *I. chrysosporus* sample (0.1 g) was hydrolyzed in 10 mL of 6 M HCl containing 5 mg/mL phenol at 110 °C for 22 h. The hydrolysate was filtered into a 50 mL volumetric flask and diluted to the 50 mL mark with deionized water. A 1.0 mL aliquot of the hydrolysate was dried at 50 °C under reduced pressure, and the residue was redissolved in 1 mL deionized water and dried again. The dried sample was subsequently dissolved in 1 mL 0.02 M HCl and passed through a 0.22 μm membrane filter for analysis using an automatic amino acid analyzer (LA8080, Hitachi, Tokyo, Japan). All analyses were conducted in triplicate.

### 2.6. Bioactive Constituent Analysis of I. chrysosporus Fruiting Bodies

Total polysaccharide content was determined using the phenol–sulfuric acid method [26]. Briefly, the *I. chrysosporus* sample (0.05 g) was mixed with 1 mL of deionized water and heated in a water bath at 100 °C for 2 h, and after centrifugation at 10,000× *g* for 10 min, the supernatant was collected. A 0.2 mL aliquot of the supernatant was combined with 0.8 mL of anhydrous ethanol. The precipitate was collected by centrifugation and dissolved in deionized water, then utilized for polysaccharide quantification, as described in [26].

Total phenolic content was measured using the Folin-Ciocalteu colorimetric assay as described in [27]. Briefly, in an alkaline nitrite medium, phenolic compounds reduce tungsten molybdic acid to form a blue compound with an absorption maximum at 760 nm, enabling the determination of total phenolic content. Samples (0.1 g) were mixed with 2.5 mL of extraction solvent (60% ethanol solution) and sonicated (300 W) at 60 °C for 30 min to extract total phenolics. Following centrifugation (12,000 rpm, 10 min), the supernatant was collected and adjusted to a final volume of 2.5 mL with the extraction solvent. Sample absorbance was measured at 760 nm using a microplate reader (Varioskan ALF, Thermo, Waltham, MA, USA). A standard curve was constructed using a 5 mg/mL (stock) gallic acid solution. Results are expressed as mg gallic acid equivalents per g dry weight (mg GAE/g DW).

Total flavonoid content in the *I. chrysosporus* sample was determined as described in [27]. Similar to the determination of total phenolic content, in an alkaline nitrite medium, flavonoids react with aluminum ions to form a red-colored chelate complex with an absorption maximum at 470 nm. Samples (0.1 g) were mixed with 1.0 mL of extraction solvent (60% ethanol solution) and sonicated (300 W) at 60 °C for 30 min. After centrifugation at 12,000 rpm for 10 min, the supernatant was collected and diluted to 1.0 mL with the extraction solvent. Absorbance was measured at 470 nm using a microplate reader (Varioskan ALF, Thermo, Waltham, MA, USA), with quantification based on a standard curve generated from dilutions of a 10 mg/mL rutin stock solution. Total flavonoid content is expressed as mg of rutin equivalents per g dry weight (mg RE/g DW).

### 2.7. Antioxidant Activity of I. chrysosporus Aqueous Extracts

Briefly, 20 g of *I. chrysosporus* sample was extracted with water (1:30, *w*/*v*) in an ultrasonic bath (200 W, 40 kHz) for 30 min at 80 °C. Following centrifugation (10,000× *g*, 10 min), the supernatant was concentrated to one-fifth of its volume by rotary evaporation under reduced pressure. Subsequently, the concentrated supernatant was mixed with ethanol at a volume ratio of 1:4, and ethanol precipitation was carried out overnight at 4 °C. The precipitate was collected by centrifugation, resuspended in deionized water, freeze-dried, and stored at −20 °C until use. Polysaccharide, protein, and polyphenol contents were determined using the methods described above. The aqueous extract was used for the determination of free radical scavenging activities using the following assays: hydroxyl, DPPH, and ferric-reducing antioxidant power (FRAP). All experiments were performed in triplicate.

The hydroxyl radical scavenging activity was determined as described in [25], with slight modifications. Briefly, 50 µL of the aqueous extract at various concentrations (0.0625, 0.125, 0.25, and 1 mg/mL) was mixed with 100 µL of 9 mM FeSO_4_ and 100 µL of 9 mM salicylic acid (dissolved in ethanol), followed by the addition of 100 µL of 8.8 mM H_2_O_2_. After incubation at 37 °C for 30 min, the absorbance of the mixture was measured at 510 nm (A_sample_). Distilled water was used as a blank (A_blank_), and another control (A_control_) was prepared by replacing H_2_O_2_ with distilled water. The hydroxyl radical scavenging activity of the aqueous extract was calculated using Equation (1):

(1) Hydroxyl radical scavenging rate (%) = [(A_control_ − A_sample_)/(A_control_ − A_blank_)] × 100%

The DPPH radical scavenging activity was determined as described in [28], with minor modifications. A 0.004% DPPH solution in methanol was prepared. Aliquots (50 μL) of the aqueous extract dissolved at various concentrations (0.0625, 0.125, 0.25, and 1 mg/mL) were mixed with 200 μL of the DPPH solution. The reaction mixture was then incubated at 37 °C for 30 min in the dark. Following centrifugation at 12,000 rpm for 5 min, the absorbance of the supernatant was measured at 517 nm (denoted as A_sample_). Ascorbic acid was used as the positive control. A control (A_control_) was prepared using methanol instead of the DPPH solution, and distilled water served as the blank (A_blank_). The DPPH radical scavenging activity of the aqueous extract was calculated using Equation (2):

(2) DPPH scavenging rate (%) = [1 − (A_sample_ − A_control_)/A_blank_] × 100%

FRAP activity was measured as described in [29]. Briefly, a FRAP working solution was prepared by mixing 0.3 mol/L acetic acid–sodium acetate buffer (pH 3.6), 0.01 M 2,4,6-Tris(2-pyridyl)-1,3,5-triazine (TPTZ) solution, and 0.02 mol/L FeCl_3_ solution at a volume ratio of 10:1:1. To 180 μL of the FRAP working solution, 5 μL of each aqueous extract sample (0.625, 1.25, 2.5, and 10 mg/mL) was added. The mixture was then incubated at 37 °C for 15 min, and absorbance was measured at 593 nm, with ascorbic acid serving as the positive control. Distilled water was used as a control in place of the sample. The regression equation for the standard curve was y = 0.2845x + 0.0002, with an R^2^ value of 0.9956. The FRAP value of each sample was calculated from the FeSO_4_ equivalent concentration corresponding to the absorbance difference (A_sample_ − A_control_) derived from the standard curve.

### 2.8. Data Analysis

Statistical analysis was performed using one-way ANOVA followed by Duncan’s multiple-range test in SPSS 21.0. Phylogenetic analysis was performed using MEGA 11 and PhyloSuite v1.2.2 to reconstruct the evolutionary relationships. The EC_50_ values were calculated using GraphPad Prism 10.1.2. The correlation heat map was generated using ChiPlot (https://www.chiplot.online/ accessed on 4 February 2026). All values are reported as the mean ± standard deviation (SD) of three independent biological replicates. A value of *p* ≤ 0.05 was considered statistically significant.

## 3. Results

### 3.1. Molecular Phylogeny

**Figure 1:**
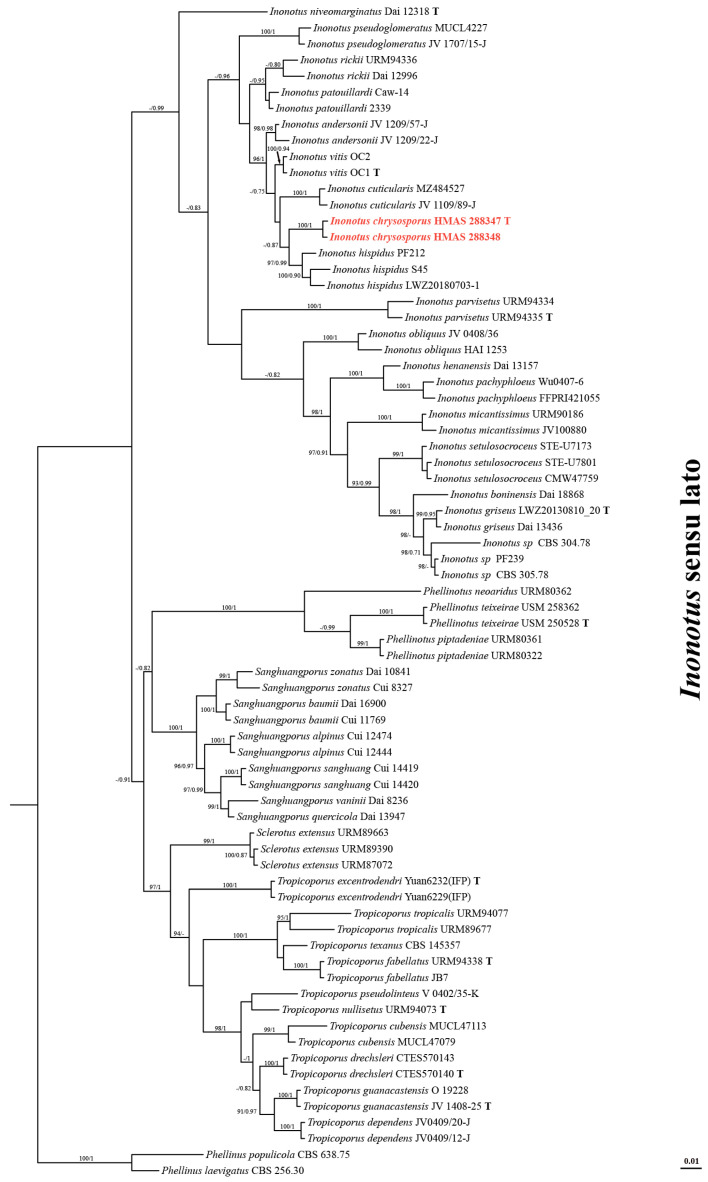
Phylogenetic tree based on concatenated ITS and nLSU sequences. Bootstrap support values (ML/BI) exceeding 90% and 0.95 are labeled on the branches. The scale bar represents substitutions per site. The novel species is marked in bold red. Arrows indicate node support values, and the ‘**T**’ signifies the type strain.

Environmental samples designated SH211 were collected as detailed in the Section 2 Materials and Methods. Fresh mycelia from the pure culture were used for total DNA extraction and PCR amplification, as well as sequencing of the nLSU and ITS loci. For the phylogenetic analyses, a total of 50 nLSU and 72 ITS sequences were downloaded from NCBI using the generated sequences as queries. A phylogenetic tree based on the concatenated ITS–nLSU dataset was constructed using sequences derived from *Phellinus populicola* and *Phellinus laevigatus* as the outgroup, as detailed in the Section 2 Materials and Methods. Bayesian and maximum likelihood phylogenetic analyses yielded nearly identical trees with no supported conflicts; therefore, only the Bayesian tree is shown (Figure 1). These analyses revealed that the isolate (subsequently designated *Inonotus chrysosporus*) represents a well-supported independent lineage, distinct from its closest known sister taxon, *I. hispidus*.

### 3.2. Taxonomy and Vegetative Incompatibility Test

**Figure 2:**
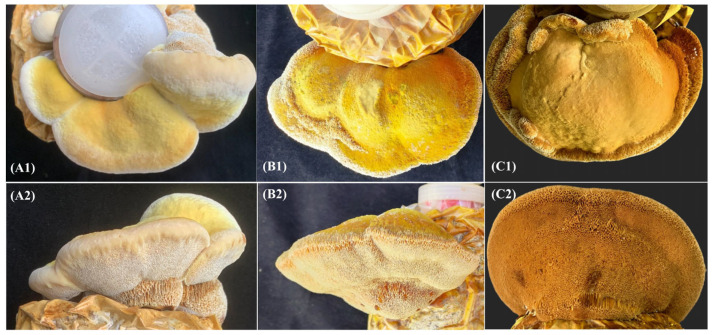
Fruiting bodies of *I. chrysosporus* at different stages: (**A1**,**A2**) early stage; (**B1**,**B2**) young and early mature stages; (**C1**,**C2**) late mature stage.

**Figure 3:**
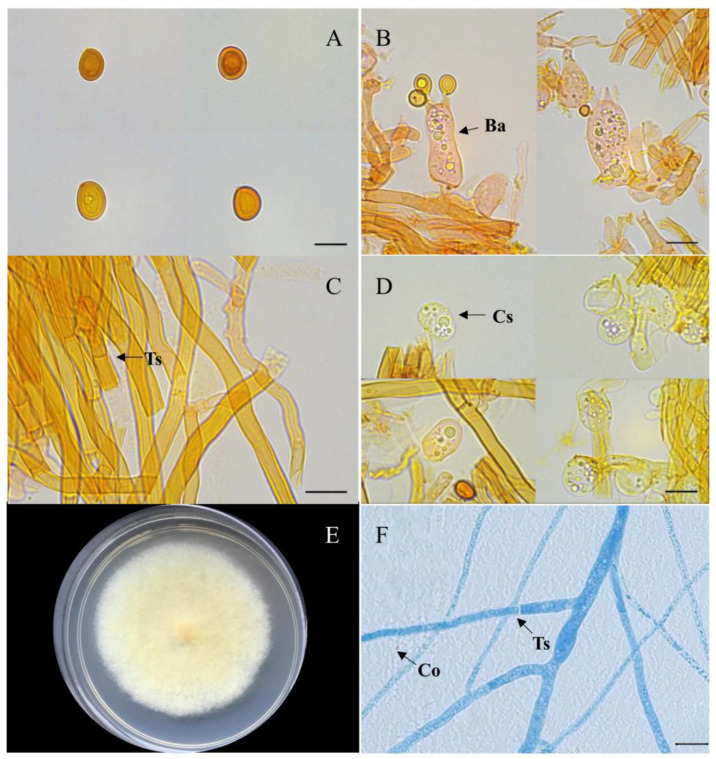
Morphological characteristics of *I. chrysosporus* D.W. Su & F.F. Song, sp. nov. (HMAS 288347): (**A**) basidiospores; (**B**) basidia and basidioles; (**C**) hyphae; (**D**) chlamydospores; (**E**) colony morphology; (**F**) mycelial morphology. Ba, basidia; Ts, transverse septa; Cs, chlamydospores; Co, conidia. All preparations were mounted in 5% KOH. Scale bars: 10 μm.

*Inonotus chrysosporus* D.W. Su & F.F. Song, sp. nov. Figure 2 and Figure 3.

MycoBank: MB 863855.

Type: CHINA. Fujian Province: Fuzhou, cultivated on a grass substrate in a plastic bag, harvested on 18 May 2025, from a mycelial culture originally derived from a single basidiospore isolate, collector: D.W. Su, HMAS 288347 (holotype).

Etymology: “chryso-” means gold, and “sporus” means spores, referring to the color of the fungal pileus and spores.

Description: *Basidiomata.* Annual, solitary or imbricate, sessile, semicircular to reniform when viewed from above. Pileus 7.53–12.58 cm broad, 5.52–6.21 cm wide from base to margin. The basidiocarp can appear as a slightly yellow (3A8) pileus with a yellowish-white to cream-colored (1A2–3A2) margin at the early stage (Figure 2(A1,A2)); a yellow to pale orange (2A6–5A7) pileus with a yellow (3A3) to brownish-orange (5C8) margin at the young and early mature stages (Figure 2(B1,B2)); and a yellowish-orange (4B7) pileus with a brownish-yellow (5C6–6C8) margin at the late mature stage (Figure 2(C1,C2)). Pileal surface tomentose to velutinate. Margin thin to thick, obtuse, hispid at the late mature stage. Context thick, spongy to fibrous. Pores circular to angular, 1–2 per mm, concolorous with the context; dissepiments entire.

Basidiospores. Ellipsoid to ovoid, yellowish, thick-walled, smooth, 7.6–11.9 × 5.5–9.3 μm, L = 10.1 μm, W = 7.9 μm, Q = 1.10–1.56 (n = 40/2) (Figure 3A). Basidia clavate, thin-walled, mostly subhyaline, 24.8–44.4 × 9.5–13.9 μm, Q = 1.81–4.69 (n = 40/2), 1.4–6.8 μm in length, with 2–4 sterigmata (Figure 3B). Hyphae, hyaline to slightly yellowish, thin- to slightly thick-walled, frequently branched, 3.6–7.4 μm in diameter (Figure 3C). Chlamydospores abundant in the context and trama, intercalary or terminal, thick-walled, smooth, globose to subglobose or ellipsoid, yellowish, 11.3–21 × 9.3–12.1 μm, Q = 1.00–2.20 (n = 40/2) (Figure 3D).

The morphological features of the colony after purification were noted (Figure 3E). The mycelium exhibited a light-yellow coloration and demonstrated rapid growth, covering the plate within approximately 8 d. The edges of the colony were densely covered with hyphae, and the aerial hyphae were vigorous, with numerous branches. The hyphae were well separated by distant transverse septa. Few conidia were visible after 8 d of growth (Figure 3F).

**Morphological distinction from *I. hispidus*.** Compared with *I. hispidus*, *I. chrysosporus* differs in its smaller basidiocarps, lighter basidiocarp color, shorter pileal hairs, absence of large pores on the hymenophore, and more ellipsoid basidiospores (Table 1).

**Figure 4:**
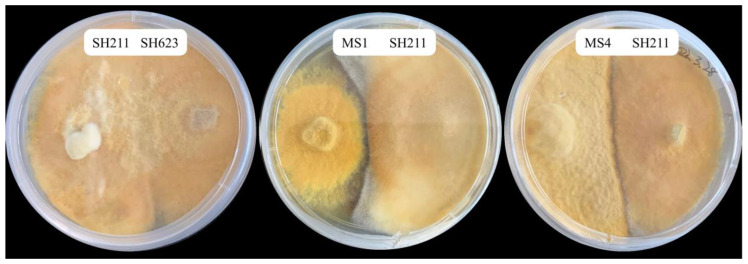
Culture plates of the vegetative compatibility assay. Compatible pairings show no demarcation line with hyphal intermingling; incompatible pairings exhibit a dark pigmented barrage zone at the junction, the clarity of which is proportional to the degree of incompatibility.

Beyond the morphological differences and phylogenetic evidence confirming their relationship, a vegetative compatibility assay revealed distinct compatibility responses. Clear vegetative incompatibility was observed between SH211 and MS1 and between SH211 and MS4, but the response between SH211 and SH623 was distinctly weaker (Figure 4).

### 3.3. Cultivation and Nutrient Analysis of I. chrysosporus Fruiting Bodies: Sawdust vs. Grass Cultivation

The *I. chrysosporus* (SH211) strain was “domesticated” by cultivation in substrate bags using a protocol detailed in the Section 2 Materials and Methods, taking ~30 d for the mycelium to fully colonize the substrate bags. During the early stage of cultivation, one or more yellow-and-white primordia emerged on the substrate surface. After the bags were sliced, the primordia grew through the opening and proceeded to mature into fruiting bodies, while golden-colored spores were released simultaneously. The average fresh weight of a mature harvested fruiting body (mushroom) was 110.5 g under sawdust cultivation and 127.7 g under grass cultivation. Fruiting bodies cultivated on sawdust substrate exhibited a denser texture than those grown on grass substrate.

The total crude protein content of the *I. chrysosporus* fruiting bodies cultivated on sawdust substrate (ICSS) and grass substrate (ICGS) was 23.40% and 22.30%, respectively, and the total fat content was 2.8% for ICSS and 3.6% for ICGS (Table 2). The ash content, an indicator of the mineral element composition of edible fungi, was 8.6% for ICSS-derived mushrooms and 9.8% for ICGS-derived mushrooms.

The *I. chrysosporus* fruiting bodies were found to contain seven essential amino acids and ten non-essential amino acids (Table 3), with growth on ICSS resulting in the highest essential amino acid content (8.66 g/100 g DW). The total amino acid (TAA) content was 19.26 g/100 g DW in ICSS and 18.01 g/100 g DW in ICGS. The ratios of essential to total amino acids (EAA/TAA) and essential to non-essential amino acids (EAA/NEAA) in mushrooms derived from ICSS were 0.45 and 0.82, whereas those in mushrooms grown on ICGS were 0.42 and 0.73, respectively (Table 3). For comparative purposes, the corresponding values for the widely cultivated medicinal mushroom *Ganoderma lucidum* [31] and chicken eggs (used as an FAO/WHO-recommended standard for amino acid scoring [32]) are also given.

Among the 17 amino acids identified in *I. chrysosporus*, the most abundant under both cultivation conditions were glutamic acid (Glu) and methionine (Met) (Table 3). Growth on ICSS resulted in higher methionine levels, whereas the mushrooms grown on ICGS contained more glutamic acid. The medicinal amino acid (MAA; Asp, Glu, Gly, Met, Leu, Phe, Tyr, Lys, and Arg) content of *I. chrysosporus* was 13.10 g/100 g DW in the ICSS group and 12.25 g/100 g DW in the ICGS group.

**Table 3:** Amino acid contents of *I. chrysosporus* fruiting bodies.

Amino Acid		Amino Acid Content (g/100 g)			
		ICSS	ICGS	*G. lucidum* [33]	Egg [32]
EAA (essential amino acid)	Thr	0.93 ± 0.01 ^a^	0.88 ± 0.01 ^b^	0.21 ± 0.01 ^d^	0.568 ^c^
	Val	1.03 ± 0.02 ^a^	1.00 ± 0.01 ^a^	0.34 ± 0.02 ^c^	0.688 ^b^
	Met *	2.58 ± 0.01 ^a^	1.74 ± 0.01 ^b^	1.38 ± 0.03 ^c^	0.357 ^d^
	Ile	0.83 ± 0.01 ^a^	0.78 ± 0.01 ^b^	0.23 ± 0.02 ^d^	0.619 ^c^
	Leu *	1.42 ± 0.02 ^a^	1.32 ± 0.01 ^b^	0.42 ± 0.02 ^d^	1.03 ^c^
	Phe *	0.84 ± 0.01 ^a^	0.77 ± 0.01 ^b^	0.22 ± 0.03 ^d^	0.612 ^c^
	Lys *	1.03 ± 0.01 ^b^	1.12 ± 0.01 ^a^	0.21 ± 0.02 ^d^	0.837 ^c^
	Trp				0.219 ^a^
NEAA (non-essential amino acid)	Asp *	1.79 ± 0.05 ^a^	1.75 ± 0.01 ^a^	0.64 ± 0.05 ^c^	1.133 ^b^
	Ser	0.94 ± 0.01 ^a^	0.86 ± 0.01 ^a^	0.27 ± 0.01 ^c^	0.854 ^b^
	Glu *	2.78 ± 0.02 ^b^	3.07 ± 0.02 ^a^	0.68 ± 0.01 ^d^	1.541 ^c^
	Gly *	0.9 ± 0.03 ^a^	0.86 ± 0.01 ^a^	0.37 ± 0.02 ^b^	0.384 ^b^
	Ala	1.16 ± 0.01 ^b^	1.18 ± 0.01 ^a^	0.37 ± 0.01 ^e^	0.639 ^c^
	Cys	0.08 ± 0.01 ^b^		0.09 ± 0.02 ^b^	0.241 ^a^
	Tyr *	0.55 ± 0.01 ^a^	0.49 ± 0.01 ^b^	0.13 ± 0.02 ^c^	0.484 ^b^
	His	0.41 ± 0.01 ^a^	0.41 ± 0.01 ^a^	0.11 ± 0.01 ^c^	0.266 ^b^
	Arg *	1.21 ± 0.01 ^a^	1.13 ± 0.01 ^b^	0.23 ± 0.02 ^d^	0.725 ^c^
	Pro	0.78 ± 0.01 ^a^	0.65 ± 0.01 ^b^	0.25 ± 0.02 ^d^	0.429 ^c^
TAA (total amino acid)		19.26 ± 0.06 ^a^	18.01 ± 0.05 ^b^	6.16 ± 0.24 ^d^	11.626 ^c^
EAA (essential amino acid)		8.66 ± 0.03 ^a^	7.61 ± 0.02 ^b^	3.01 ± 0.12 ^d^	4.711 ^c^
NEAA (non-essential amino acid)		10.60 ± 0.02 ^a^	10.40 ± 0.04 ^b^	3.15 ± 0.12 ^d^	6.92 ^c^
MAA (medicinal amino acid)		13.10 ± 0.03 ^a^	12.25 ± 0.04 ^b^	4.29 ± 0.17 ^d^	7.10 ^c^
EAA/TAA (%)		44.96 ± 0.03 ^b^	42.25 ± 0.12 ^c^	48.92 ± 0.17 ^a^	42.41 ^c^
EAA/NEAA (%)		81.70 ± 0.39 ^b^	73.17 ± 0.36 ^c^	95.76 ± 0.66 ^a^	73.62 ^c^
MAA/NEAA (%)		68.02 ± 0.06 ^b^	68.02 ± 0.07 ^b^	69.64 ± 0.23 ^a^	61.10 ^c^

* Also belongs to medicinal amino acids. Different lowercase superscript letters denote statistically significant differences (*p* < 0.05).

### 3.4. Bioactive Constituents of I. chrysosporus Fruiting Bodies in Different Substrates

**Figure 5:**
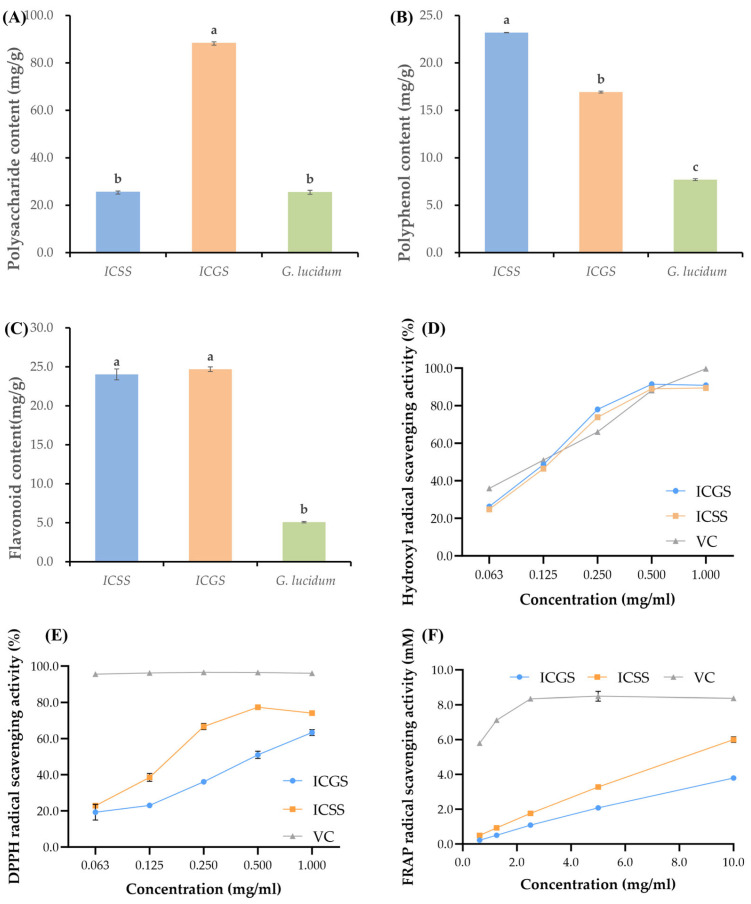
Variations in bioactive constituents of *I. chrysosporus* and the antioxidant activities of its aqueous extracts: (**A**) polysaccharide contents; (**B**) total phenolic contents; (**C**) flavonoid contents; (**D**) hydroxyl free radical scavenging activity; (**E**) DPPH free radical scavenging activity; (**F**) ferric-reducing antioxidant power (FRAP). Different lowercase letters indicate significant differences (*p* < 0.05). Data on *G. lucidum* were obtained from [34].

The total polysaccharide content of *I. chrysosporus* mushrooms was determined as detailed in the Section 2 Materials and Methods. Growth on ICGS yielded the highest polysaccharide content at 88.5 mg/g DW, significantly exceeding the levels in the ICSS group (25.7 mg/g DW) and in *G. lucidum* (25.6 mg/g DW) (Figure 5A). The total phenolic and flavonoid contents were quantified and expressed as gallic acid (GAE) and rutin (RE) equivalents, respectively, in milligrams per gram of sample. The total phenolic content was significantly higher in *I. chrysosporus* mushrooms, measuring 16.93 mg/g (ICGS) and 23.20 mg/g (ICSS), corresponding to increases of 2.20-fold and 3.01-fold compared to *G. lucidum*, respectively (Figure 5B). The flavonoid content of *I. chrysosporus* was also significantly higher than that of *G. lucidum* (5.08 mg/g) [34], representing increases of approximately 4.86-fold (ICGS) and 4.72-fold (ICSS), respectively (Figure 5C).

The aqueous extracts of *I. chrysosporus* mushrooms derived from either cultivation condition showed strong antioxidant activity (Figure 5D–F). A chemical composition analysis of the *I. chrysosporus* aqueous extracts indicated that their main constituents were polysaccharides, with minor amounts of glycoproteins and polyphenols (Table 4). A concentration-dependent increase in hydroxyl radical scavenging activity was observed for the aqueous extracts of *I. chrysosporus*, with efficacy comparable to that of ascorbic acid (VC). When the concentration of *I. chrysosporus* aqueous extracts reached 0.50 mg/mL, the scavenging percentages peaked at 90.83 ± 0.17% (ICGS) and 89.43 ± 0.12% (ICSS) (Figure 5D). The half-maximal effective concentrations (EC_50_) of *I. chrysosporus* aqueous extracts were 0.149 ± 0.008 (ICGS) and 0.151 ± 0.004 (ICSS) mg/mL, respectively.

The DPPH radical scavenging activity of the *I. chrysosporus* aqueous extracts exhibited considerable variation (Figure 5E). At a concentration of 1 mg/mL, the scavenging activity of the ICGS extract was 63.35 ± 1.62%, whereas that of the ICSS extract was 74.10 ± 0.48%. The EC_50_ values of the *I. chrysosporus* aqueous extracts were 0.358 ± 0.072 (ICGS) and 0.155 ± 0.012 (ICSS) mg/mL.

Ferric-reducing antioxidant power (FRAP) assays revealed that the *I. chrysosporus* aqueous extracts exhibited potent reducing activity in a dose-dependent manner over the concentration range of 0.625 to 10.00 mg/mL. The highest value was observed at the maximum concentration of 10.00 mg/mL (Figure 5F). The aqueous extract of *I. chrysosporus* cultivated on sawdust substrate showed high reducing activity of 6.00 mM, whereas that of mushrooms cultivated on grass substrate was 3.79 mM.

**Table 4:** Component analysis of *I. chrysosporus* aqueous extracts.

Sample	Polysaccharides (mg/g)	Glycoproteins (mg/g)	Polyphenols (mg/g)
ICGS	695.14	21.89	18.06
ICSS	482.13	42.28	104.56

**Figure 6:**
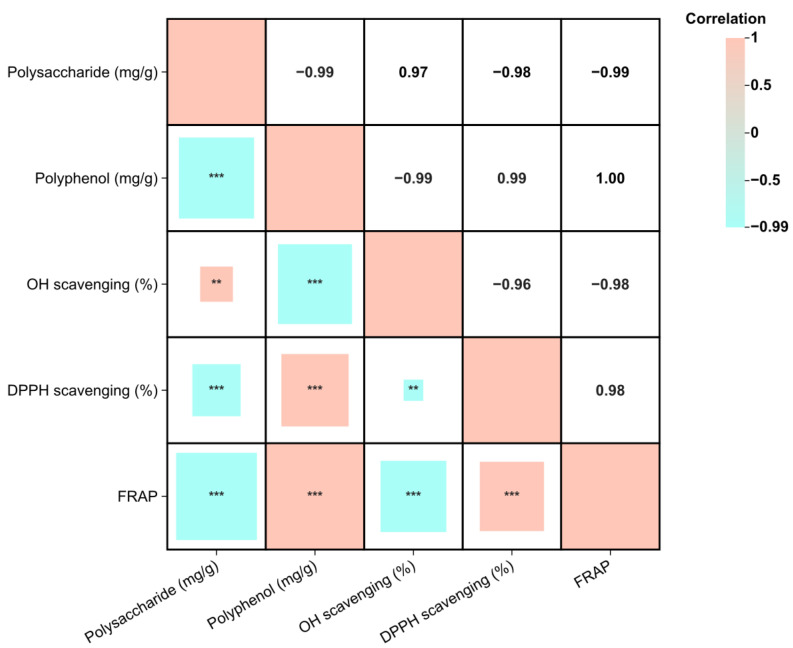
Correlation heatmap of bioactive constituents and antioxidant activities. ** *p* < 0.01, *** *p* < 0.001.

To investigate how antioxidant activity relates to the bioactive constituents of *I. chrysosporus*, correlations between each pair of indicators were analyzed using ChiPlot (Figure 6). The results suggest that polysaccharide content is positively associated with hydroxyl radical scavenging activity but negatively correlated with DPPH radical scavenging activity and FRAP. Furthermore, polyphenol content is strongly associated with DPPH radical scavenging activity and FRAP.

## 4. Discussion

The medicinal value of a wide range of mushrooms, including *Ganoderma*, *Inonotus*, and a myriad of others, has long been recognized. *Inonotus* is a polyphyletic genus that includes more than 100 species, many of which are of ecological importance and are used in traditional medicine, e.g., *I. obliquus* and *I. hispidus* [1,35]. *I. hispidus* has been used for millennia as part of Chinese, and likely other, pharmacopeias [4]. During collection, we identified a “shaggy bracket-like” mushroom, which, after in vitro cultivation and phylogenetic and morphological analyses, we designated *I. chrysosporus*. Both molecular phylogenetic and morphological analyses indicated that *I. chrysosporus* is closely related to, but distinct from, *I. hispidus*. Vegetative incompatibility assays were somewhat inconclusive, suggesting either recent divergence or some level of compatibility. This phenomenon is consistent with the report on *Inonotus cerradensis* [35], in which different strains of the same species were found to be either fully compatible or to exhibit only a weak barrage line. It is noteworthy that antagonism was absent or weak among closely related strains but clearly evident between distantly related ones—a pattern that aligns well with the traditional view of antagonism as a measure of genetic relatedness.

As a sister species of *I. hispidus*, *I. chrysosporus* sp. nov. may possess biological activities like those of *I. hispidus*. However, how mushrooms are cultivated, i.e., the growth substrate, has long been recognized as affecting their biological activity, with significant gaps remaining in our understanding of this activity. We therefore sought to determine how growth substrate influences the growth and bioactive properties of *I. chrysosporus* mushrooms.

Mushrooms are widely recognized for their high protein content and notably low fat content, making them a low-calorie food [36]. To assess the nutritional quality of our target species against established references, *G. lucidum* was selected as the fungal comparator because of the extensive compositional data available, while chicken eggs were included as the FAO/WHO-recommended protein standard for amino acid scoring. Remarkably, the total crude protein content of the *I. chrysosporus* isolate (22.30–23.40%) exceeded that of *G. lucidum* (11.70%), another widely used medicinal mushroom [31]. The total crude protein content was even higher than that of chicken eggs (13.30%) [32], while the fat content was considerably lower (2.8–3.6% vs. 8.6%, respectively) [32]. Mushrooms contain significant levels of various vitamins, including B-complex vitamins (B1, B2, B3, B9, and B12), ascorbic acid (vitamin C), ergocalciferol (vitamin D2), and tocopherol (vitamin E), alongside a broad spectrum of macro- and trace minerals such as potassium, phosphorus, iron, zinc, copper, selenium, magnesium, sodium, calcium, molybdenum, cobalt, titanium, and cadmium [37]. After high-temperature calcination, the remaining inorganic material is referred to as ash, which primarily consists of inorganic salts and oxides. Ash content can serve as an indicator of the mineral element composition of edible fungi. The ash content of the *I. chrysosporus* isolate (8.6–9.8%) was significantly higher than that of *G. lucidium* (2.31%) [31] and eggs (0.9%) [32]. These data indicate that *I. chrysosporus* has a rich mineral profile, which may contribute to meeting daily human trace element requirements. Overall, *I. chrysosporus* represents a high-protein, mineral-dense, and low-fat food source that aligns with the nutritional criteria for a healthy diet. Furthermore, the nutritional profile of the fruiting bodies of the wild strain was influenced by the cultivation substrate used. The fat and ash contents were highest in the fruiting bodies grown on grass substrate, while the protein content was highest in those grown on sawdust substrate.

Beyond their role as the building blocks of protein, amino acids are crucial for many physiological processes. The amino acid profile of *I. chrysosporus* revealed that glutamic acid (Glu) and methionine (Met) were the most abundant amino acids. Glutamic acid, one of the flavor-enhancing amino acids, serves as both the primary umami stimulus and an integral component of nitrogen metabolism and protein synthesis [25]. Beyond its fundamental role as an EAA, methionine (a medicinal amino acid) deficiency has been implicated in pathologies including liver cirrhosis, fatty liver, and cardiovascular disease, while also enhancing antioxidant defenses and modulating cellular functions [38]. Medicinal amino acids are necessary for maintaining nitrogen balance in the human body, yet they are present in only small amounts in common plant-based foods [39]. And nearly half of them are essential amino acids (EAAs), which cannot be synthesized by the human body. Our data show that growth of *I. chrysosporus* on either substrate resulted in high ratios of MAA to total amino acids (MAA/TAA ≈ 68.02%), which is comparable to that of *G. lucidum* (69.64%) and higher than that of chicken eggs (61.10%) [32,33]. Furthermore, both the EAA/TAA and EAA/NEAA-ratios exceeded the ideal standards recommended by FAO/WHO (0.40 and 0.60, respectively) [40].

Polysaccharides are often critical components affecting the bioactivity of edible fungi [14,25]. Cultivation on the grass substrate resulted in a 2.44-fold increase in polysaccharide content compared to that cultivated on the sawdust substrate. The bioactivity of polysaccharides is determined by their structural properties, including molecular weight, monosaccharide composition, and the types of glycosidic linkages [41]. Since the structure of edible fungal polysaccharides is influenced by the growth substrate, previous studies have explored the addition of exogenous substances to enhance polysaccharide profiles [42]. Here, we demonstrate that the grass substrate significantly increased the polysaccharide content of *I. chrysosporus.* However, whether this increase also alters its polysaccharide structure and, potentially, its bioactivity requires further investigation.

Polyphenols are aromatic hydroxyl derivatives that are widely produced through the secondary metabolism of higher fungi. The hydroxyl groups on the benzene ring enable polyphenols to act as electron donors, participating in various reactions that confer diverse biological properties, including antioxidant and antitumor activities [43,44]. Gallic, caffeic, and p-coumaric acids have been reported as the predominant phenolic compounds in mushrooms [45]. Flavonoids are a class of low-molecular-weight polyphenolic metabolites widely distributed in plants and higher fungi. The flavonoid constituents of medicinal mushrooms primarily comprise myricetin, rutin, naringenin, quercetin, morin, and hesperetin [46]. Our results demonstrate that *I. chrysosporus* is rich in polyphenols and flavonoids, suggesting strong antioxidant potential and associated health-promoting effects.

To verify this, we further assessed the antioxidant activity of extracts derived from the fungus. Our results show that the hydroxyl radical scavenging activity of *I. chrysosporus* under different cultivation conditions is robust and stable, which is consistent with reports on the related *Sanghuangporus* fungi [47], in which the maximum scavenging activities of *S. baumii*, *S. vaninii*, and *S. sanghuang* were determined to be 90.08%, 87.61%, and 94.06%, respectively. The DPPH radical scavenging activity of *I. chrysosporus* cultivated on sawdust substrate was greater than that of mushrooms cultivated on grass substrate. Our results are consistent with those reported for *Sanghuangporus* fungi, in which the maximum DPPH radical scavenging activity was determined to be ~72% [48].

Collectively, our findings indicate that *I. chrysosporus* cultivated under different conditions exhibits potent antioxidant activity, comparable to or greater than that of other *Sanghuangporus* species or even ascorbic acid. This underscores the potential of *I. chrysosporus* as a high-value cultivated mushroom. The bioactive and antioxidant constituents of mushrooms are broadly categorized into two major classes: high-molecular-weight compounds (e.g., polysaccharides) and low-molecular-weight compounds (e.g., polyphenols, flavonoids, and catechols) [49]. In this study, the antioxidant activity of the aqueous extracts of *I. chrysosporus* showed some variation but was generally high. The aqueous extracts of *I. chrysosporus* cultivated on sawdust substrate exhibited higher polyphenol and flavonoid contents, stronger DPPH radical scavenging activity, and higher FRAP values. The *I. chrysosporus* cultivated on grass substrate yielded a greater polysaccharide content and stronger hydroxyl radical scavenging activity. Overall, important variations in the profiles of active constituents and antioxidant capacities were observed in *I. chrysosporus* grown under different cultivation conditions, highlighting the cultivation substrate as one of the key determinants of its bioactive properties, although high levels of activity were observed under both growth conditions.

## 5. Conclusions

This study systematically compared the effects of different cultivation substrates on the accumulation of bioactive components and the biological activities of an environmental (wild) isolate (SH211), which was identified as a novel *Inonotus* species based on morphological and molecular analyses. Although important differences in nutrient components (protein, amino acids, fat, and ash) and bioactive constituents (polysaccharides, polyphenols, and flavonoids) were observed in the *I. chrysosporus* strain grown on two distinct substrates, our results indicate that this strain is a high-protein, mineral-rich, and low-fat nutrient source, with abundant and diverse essential amino acids. The aqueous extracts derived from *I. chrysosporus* possessed potent in vitro antioxidant activity, effectively scavenging OH and DPPH radicals and exhibiting strong total antioxidant capacity. This suggests that *I. chrysosporus* could serve as a promising source of natural antioxidants. In addition, cultivation on grass substrate offered a substantial advantage for promoting the targeted production of polysaccharides.

Our data provide a foundation for further investigations into the bioactivity of *I. chrysosporus* and practical guidance for refining its artificial cultivation strategies, ultimately contributing to the development and utilization of *I. chrysosporus* for human health applications.

## Figures and Tables

**Table 1:** Comparison of the characteristics of *Inonotus chrysosporus* and *Inonotus hispidus*.

Characteristics	Basidiocarp				Basidiospores (μm)
	Size (cm)	Color (Late Mature Stage)	Pileus	Hymenophore (Large Pores)	
*I. chrysosporus*	7.53–12.58 × 5.52–6.21	yellowish-orange, brownish-yellow	tomentose to velutinate	absent	7.6–11.9 × 5.5–9.3
*I. hispidus* [30]	16–25 × 6–12	black, dark brown, blackish, blackish-blue	suede-like to hispidus	present, scattered	9.5–10.3 × 8.6–10

**Table 2:** Nutrient contents of *I. chrysosporus* fruiting bodies.

Nutrient Composition	Content (g/100 g)			
	ICSS	ICGS	*G. lucidum* [31]	Egg [32]
crude protein	23.40 ± 0.04 ^a^	22.30 ± 0.03 ^b^	11.70 ± 0.35 ^d^	12.04 ^c^
crude fat	2.80 ± 0.02 ^c^	3.60 ± 0.16 ^b^	1.26 ± 0.09 ^d^	8.60 ^a^
total ash	8.60 ± 0.01 ^b^	9.80 ± 0.03 ^a^	2.31 ± 0.12 ^c^	0.90 ^d^

Different lowercase superscript letters denote statistically significant differences (*p* < 0.05).

## Data Availability

All newly generated sequences were deposited in GenBank (https://www.ncbi.nlm.nih.gov/genbank/ accessed on 4 February 2026). All new taxa were linked with MycoBank (https://www.mycobank.org/ accessed on 4 February 2026).

## Appendix A

**Table A1:** Detailed specimen information and GenBank accession numbers.

Species	Voucher Specimen No.	GenBank No.	
		ITS	nLSU
*Inonotus andersonii*	JV 1209/57-J	KF446593	
*Inonotus andersonii*	JV 1209/22-J	KF446592	
*Inonotus boninensis*	Dai 18868	MZ484601	OL413400
*Inonotus cuticularis*	MZ484527	MZ484527	
*Inonotus cuticularis*	JV 1109/89-J	KF446595	
*Inonotus griseus*	Dai 13436	KX364802	KX364823
*Inonotus griseus* T	LWZ20130810_20	KM434333	KX890461
*Inonotus henanensis*	Dai 13157	KP030783	KX832918
*Inonotus hispidus*	S45	EU282482	EU282484
*Inonotus hispidus*	PF212	GU068592	
*Inonotus hispidus*	LWZ20180703-1	MT332137	ON063858
*Inonotus micantissimus*	URM90186	MG576057	MG576125
*Inonotus micantissimus*	JV100880	JF692194	
*Inonotus niveomarginatus* T	Dai 12318	KC456245	
*Inonotus obliquus*	HAI 1253	GQ253463	
*Inonotus obliquus*	JV 0408/36	KF446605	OL413406
*Inonotus pachyphloeus*	Wu0407-6	KP030785	KP030770
*Inonotus pachyphloeus*	FFPRI421055	LC822062	LC822097
*Inonotus parvisetus*	URM94334	MT908374	MT906640
*Inonotus parvisetus* T	URM94335	MT908372	MT906638
*Inonotus patouillardi*	Caw-14	GQ249883	
*Inonotus patouillardi*	2339	AY072024	
*Inonotus pseudoglomeratus*	JV 1707/15-J	MN318437	
*Inonotus pseudoglomeratus*	MUCL4227	MZ484608	
*Inonotus rickii*	Dai 12996	KC479128	MH101019
*Inonotus rickii*	URM94336	MT908373	MT906639
*Inonotus setulosocroceus*	STE-U7801	KP279294	
*Inonotus setulosocroceus*	CMW47759	MH599090	MH599115
*Inonotus setulosocroceus*	STE-U7173	KP279293	
*Inonotus* sp.	CBS 304.78	HM362907	
*Inonotus* sp.	CBS 305.78	GU111923	
*Inonotus* sp.	PF239	GU111924	
*Inonotus* sp.	HMAS 288347	PZ413114	PZ413120
*Inonotus* sp.	HMAS 288348	PZ413115	PZ413121
*Inonotus vitis*	OC2	MN108119	MN113945
*Inonotus vitis* T	OC1	MN108118	MN113944
*Phellinotus neoaridus*	URM80362	KM211294	KM211286
*Phellinotus piptadeniae*	URM80322	KM211290	KM211282
*Phellinotus piptadeniae*	URM80361	KM211288	KM211280
*Phellinotus teixeirae*	USM 258362	MZ954854	MZ964975
*Phellinotus teixeirae* T	USM 250528	MZ954853	MZ964972
*Phellinus laevigatus*	CBS:256.30	MH855136	MH866584
*Phellinus populicola*	CBS:638.75	MH860960	MH872729
*Sanghuangporus alpinus*	Cui 12444	MF772782	MF772800
*Sanghuangporus alpinus*	Cui 12474	MF772783	MF772801
*Sanghuangporus baumii*	Dai 16900	MF772785	MF772802
*Sanghuangporus baumii*	Cui 11769	MF772784	MF772803
*Sanghuangporus quercicola*	Dai 13947	KY328309	MF772809
*Sanghuangporus sanghuang*	Cui 14419	MF772789	MF772810
*Sanghuangporus sanghuang*	Cui 14420	MF772790	MF772811
*Sanghuangporus vaninii*	Dai 8236	MF772791	MF772812
*Sanghuangporus zonatus*	Dai 10841	JQ860306	KP030775
*Sanghuangporus zonatus*	Cui 8327	JX069837	MF772817
*Sclerotus extensus*	URM89390	MN795108	MN812244
*Sclerotus extensus*	URM87072	MN795109	MN812245
*Sclerotus extensus*	URM89663	MN795110	MN812246
*Tropicoporus cubensis*	MUCL47079	JQ860325	KP030776
*Tropicoporus cubensis*	MUCL47113	JQ860324	
*Tropicoporus dependens*	JV0409/12-J	KC778777	MF772818
*Tropicoporus dependens*	JV0409/20-J	KC778778	
*Tropicoporus drechsleri*	CTES570143	MG242436	MG242443
*Tropicoporus drechsleri* T	CTES570140	MG242439	MG242444
*Tropicoporus excentrodendri*	Yuan6229(IFP)	KP030789	
*Tropicoporus excentrodendri* T	Yuan6232(IFP)	KP030790	
*Tropicoporus fabellatus*	JB7	MT925653	MT925654
*Tropicoporus fabellatus* T	URM94338	MT908376	MT906643
*Tropicoporus guanacastensis*	O 19228	KP030794	MF772819
*Tropicoporus guanacastensis* T	JV 1408-25	KP030793	KP030778
*Tropicoporus nullisetus* T	URM94073	MN795129	MN812261
*Tropicoporus pseudolinteus*	V 0402/35-K	KC778781	MF772820
*Tropicoporus texanus*	CBS 145357	MN108124	MN113950
*Tropicoporus tropicalis*	URM89677	MN795112	MN812248
*Tropicoporus tropicalis*	URM94077	MN795113	MN812249
